# Ficus-mediated green synthesis of manganese oxide nanoparticles for adsorptive removal of malachite green from surface water

**DOI:** 10.1007/s11356-022-24199-8

**Published:** 2022-11-17

**Authors:** Ibrahem Mohamed Abouzeid Hasan, Hassan M. A. Salman, Olfat M. Hafez

**Affiliations:** Chemistry Department, Faculty of Science, South Valley University, Qena, 83523 Egypt

**Keywords:** Adsorption, Ficus, Green synthesis, Malachite green, Manganese oxide nanoparticles, Removal

## Abstract

The extract of ficus leaves was used to prepare manganese (IV) oxide nanoparticles (MnO_2_ NPs) for the first time. Several different analytical techniques were used to characterize the prepared MnO_2_ NPs. MnO_2_ has spherical crystals that are ~ 7 nm on average in size and have 149.68 m^2^/g of surface area and 0.91 cm^3^/g of total pore volume. Malachite green (MG) dye was then taken out of the water by adsorption using MnO_2_ NPs. Optimization of various adsorption parameters resulted in 188.68–277.78 mg/g maximum adsorption capacities at 298–328 K tested temperatures and 99.6% removal of 50 mg/L MG within 90 min using MnO_2_ dose of 0.01 g at pH 10 and 298 K. The results were tested using pseudo-first order, pseudo-second order, intraparticle diffusion, Elovich, and Liquid film kinetic models as well as Langmuir, Freundlich, Temkin, and Dubinin-Radushkevich isotherm models. The most likely models to describe the adsorption process at 298 K are pseudo-second-order kinetics (*R*^2^ = 0.997) with a rate constant of 4 × 10^−4^ g/(mg.min) and Langmuir isotherm (*R*^2^ = 0.973). Additionally, the positive values of enthalpy change (3.91–67.81 kJ/mol) and the negative values of Gibb’s free energy (− 3.38 to − 19.7 kJ/mol) indicate that the process is endothermic, spontaneous, and thermodynamically feasible. MnO_2_ NPs sustained their adsorption efficiency at 90.4% after 5 sorption cycles. MnO_2_ appears to be more selective for MG in studies examining the adsorption of various cationic dyes. Lately, the biosynthesized MnO_2_ NPs can be utilized to remove MG from aqueous solutions effectively.

**Supplementary Information:**

The online version contains supplementary material available at 10.1007/s11356-022-24199-8.

## Introduction

Contamination of drinking water is a major concern for people all around the world. Dye wastewater, however, is the most important sort of effluent to deal with. As printing and dying became more industrialized in the twentieth century, many dyes were released into the aquatic environment. Dyes are used to color a wide range of materials, including printing paper, textiles, leather, plastics, and rubber (Dutta et al. 2021; Nithya et al. 2021; Pai et al. 2021; Tan et al. 2015; Chowdhury et al. 2011). Ten thousand different dyes and pigments are used in the industry, with 700,000 tonnes of dyes and pigments produced each year. Dyes can cause a wide range of health issues, including problems with the central nervous system, brain, reproductive organs, liver, and kidneys (Zhou et al. 2019a, b). Therefore, removing dyes from wastewater is an intractable problem that researchers have faced so far.

Malachite green (MG) is a triphenyl methane cationic dye which is used in dying several types of fibers and materials. It is also used in fish farming to treat parasites, fungal infections, and bacterial infections (Altintig et al. 2021; Nethaji et al. 2010). Despite its wide range of uses, MG toxic properties make it dangerous to aquatic life and human health when present in water. Besides causing damage to organ systems such as the heart, liver, and kidneys, it also has teratogenic properties and can lead to lesions of the skin, eye, lung, and bone tissues (Swan and Zaini 2019; Chowdhury et al. 2011). Therefore, it is important to remove MG dye before wastewater discharge into the aquatic environment.

Numerous strategies have been utilized throughout the years to remove colors from dyeing effluent. These methods incorporate membrane filtration (Zhou et al. 2019a, b), ion exchange (Ma et al. 2019), advanced oxidation processes (Barbosa et al. 2019; Cho et al. 2022), photocatalytic degradation (Mostafa and Amdeha 2022), and adsorption (Yousefi et al. 2021; Zaidi et al. 2019). Adsorption is the method of choice for removal of inorganic and organic contaminants from the environment due to its wide range of applications, simplicity of design, ease of use, low cost, high efficiency, and the capacity to regenerate used materials (Choudhary et al. 2020). Many of the applied sorbents in the literature such as clay, activated carbon, zeolites, and husks have low adsorption capacity and stability. But, metal oxide nanoparticles are usually good adsorbents due to their high stability, inexpensive manufacturing methods, high surface area, and low toxicity (Nikolova and Chavali 2020). Therefore, more reseach is required in the field of synthesis and application of metal oxide nanoparticles as adsorbents for environmental remediation.

Among several metal oxides such as Ag_2_O, TiO_2_, ZnO, MoO_3_, ZrO_2_, WO_3_, α-Fe_2_O_3_, and SnO_2_, manganese oxides are particularly interesting owing to their physical and chemical features. Manganese oxides have many forms, but the most well-known and stable are Mn_3_O_4_, Mn_2_O_3_, and MnO_2_ (Silva et al. 2021; Sun et al. 2021). They can be used in many fields such as ion exchange, biosensors, energy storage, and adsorption (Siddique et al. 2021). Owing to their availability and eco-friendliness, manganese oxides and their synthesis techniques have drawn considerable attention. Wet techniques are the most extensively employed of all the known procedures (Behzad et al. 2021). These can be further divided into three categories: physical, chemical, and biological techniques (Abdelgawad et al. 2017). Physical and chemical methods have disadvantages such as low efficiency, high cost, and environmental pollution, which result in the challenge of large-scale production of some nanoparticles (Zhang et al. 2022). Alternatively, biosynthesis using plants, bacteria, fungi, and algae often provides several benefits, such as being very simple, inexpensive, safe, and environmentally (Zhang et al. 2022). In the latter case, the utilization of plant components for nanoparticle synthesis is better than other methods since it is environmentally beneficial, economical, and straightforward (Jadoun et al. 2021). In addition, plant-mediated synthesis tends to be faster than microorganisms, is more cost-effective, and is relatively easy to scale up for the production of large quantities of nanoparticles (Shah et al. 2015).

Manganese oxide nanoparticles were biosynthesized utilizing different plant extracts including *Aloe vera* (Joshi et al. 2020), *Viola betonicifolia* (Lu et al. 2021), *Kalopanax pictus* (Babaei et al. 2021), *Matricaria chamomilla* L. (Ogunyemi et al. 2019), *Euphorbia heterophylla* L. (Dewi and Yulizar 2020), orange’s peel and juice (Hashem et al. 2018), lemon juice (Manjula et al. 2020), and *Bryophyllum pinnatum* leaves (Ullah et al. 2020)*.*

*Fi*c*us benjamina* (FB) (family: *Moraceae*) also known as the weeping fig is a Southeast Asian evergreen tree. Several portions of this plant have been employed in traditional medicine to treat anti-dysentery and skin ailments (Jassal and Sharma 2019). Leaf extracts of FB tree showed a richness of bioactive chemical compounds including phenolic mixtures, carbohydrates, saponins, flavonoids, alkaloids, proteins, and tannins (Kobelnik et al. 2021). According to our literature search results, FB was utilized for the biosynthesis of silver (Puente et al. 2019), iron/copper (Abdel-Aziz et al. 2020), and iron (Abdel-Aziz and Fayyadh 2021) nanoparticles. However, no research article has been published on the use of FB for MnO_2_ NPs biosynthesis.

Therefore, the goal of this research is the green synthesis of MnO_2_ NPs using phytochemicals in FB leaves as reducing and capping agents for the first time. The resulting MnO_2_ is fully characterized and then applied for efficient adsorption of MG, as a model dye, from aqueous solution. The agitation time, pH, MnO_2_ dose, MG concentration, temperature, and ionic strength were investigated as operating parameters influencing the MG adsorption procedure. Various adsorption isotherms, kinetics, and thermodynamics were examined and reported. In addition, selectivity, regeneration, reusability, and stability were assessed. Finally, the MG adsorption mechanism by MnO_2_ NPs was proposed.

## Materials and procedures

### Materials

Merck Co. in Germany provided the potassium permanganate (KMnO_4_, 99.9% pure). The green production of MnO_2_ nanoparticles was made possible by the aqueous extract of FB leaves. Sigma-Aldrich Chemicals in the USA provided the model organic pollutant MG dye (purity 98%). The remaining chemicals were of analytical grade and were utilized as received. At room temperature, all aqueous solutions were prepared using bidistilled H_2_O.

### FB leaves’ extract (FBLE)

With few modifications, the aqueous leaf extract was prepared by a previous study (Hasan et al. 2021). Briefly, 250 mL of bidistilled water was used to simmer 25 g of newly cleaned FB leaves for 2 h at 80 °C, and then the mixture was steeped overnight. The mixture was then decanted and twice filtered via Whatman filter paper (125 mm). It was then time to collect and store the pale brown, transparent solution containing the polyphenolic compounds for future use.

### Biosynthesis of MnO_2_ NPs using FBLE

Five grams of KMnO_4_ were dissolved in 100 ml BDS, acidified with dilute H_2_SO_4_, and then added to FBLE in a 1:2 volume ratio. A magnetic stirrer was used to vigorously agitate the mixture for three hours at 70 °C, resulting in the change of the purple color of permanganate (Mn^7+^) to dark brown color and the formation of a black precipitate of MnO_2_ NPs (Mn^4+^). To ensure complete manganese ion reduction, the mixture was aged overnight before being dried overnight at 120 °C (Hasan et al. 2022). The remaining residue was gathered, rinsed three times with bidistilled H_2_O and ethanol, and dried overnight in an oven at 80–90 °C. As a final step, the black substance was ground into a fine powder using a pestle and mortar and kept in an airtight container.

### Adsorption experiments

Adsorption studies on MnO_2_ NPs were conducted in batches, allowing for a full examination of all factors that affect the adsorption process. In this technique, a consistent mass of MnO_2_ NPs was mixed with a specified amount of MG solution and swirled at room temperature (25 °C). The adsorption duration, MnO_2_ NPs dose, pH, MG concentration, temperature, interfering ions, and reusability have all been examined in this study. It was decided to use UV–vis spectroscopy to determine the MG concentrations in the centrifuged samples. The difference between the MG concentrations at the beginning and end of the experiment was utilized to compute the adsorption concentration of MG. Equation (1) is used to calculate the removal efficiencies *R%*:

1 $$R\%=\left(\left({C}_{o}-{C}_{f}\right)/{C}_{o}\right)*100$$

Then, the adsorption capacity *q*_e_ is calculated from Eq. (2)):

2 $${q}_{e}=\left({C}_{o}-{C}_{f}\right)\times \left(V/W\right)$$

where the initial and final concentrations of MG are *C*_o_ and *C*_f_ (mg/L), the volume of the treated solution is *V* (L), and the weight of MnO_2_ NPs is *W* (g).

#### Optimization of adsorption experiments

Various parameters impacting the adsorption process were tested one by one including solution pH, MnO_2_ NPs dose, agitation time, MG dye concentration, temperature, interfering ions, and the possibility of reusing the adsorbent.

A series of tests were performed within pH range from pH 3 to pH 10 to investigate the optimum pH which can achieve the best removal efficiency of MG by MnO_2_ NPs. This was accomplished by mixing 10 mg of MnO_2_ NPs with 50 mL of MG aqueous solution of fixed concentration 10 mg/L for 15 min at 25 °C. The pH values were adjusted by 0.1 M NaOH and 0.1 M HCl solutions.

The impact of the adsorbent mass on the MG adsorption was examined by direct mixing of 50 mL MG solution (10 mg/L) with different MnO_2_ NPs masses (1 mg, 2.5 mg, 5 mg, 7.5 mg, and 10 mg) for 15 min at pH 10 and 25 °C.

The effect of agitation time on MG adsorption was tested by mixing 50 mL MG aqueous solutions (50 mg/L) with 10 mg of MnO_2_ NPs at different time intervals ranged from 15 to 90 min. The other parameters were fixed at pH 10 and temperature 25 °C.

A set of experiments were conducted with 50 mL solutions of different MG concentrations (from 10 to 50 mg/L) and agitated with fixed sorbent dose of 10 mg at constant temperature (25 °C) and pH 10. After that, MG samples were filtered off and UV–vis measured, and the final MG concentration was determined.

Adsorption thermodynamic studies were carried out at four temperatures of 298, 308, 318, and 328 K. In each set, 50 mL of MG solution (50 mg/L) and adsorbent dose 10 mg were stirred for 90 min at pH 10.

The interference of NaCl and CaCl_2_ (concentration 2–10 g/L) was also studied at optimum conditions: MnO_2_ NPs dose of 10 mg, MG initial concentration of 50 mg/L, agitation time of 90 min, pH 10, and temperature 25 °C.

Two vital parameters in the realistic and large-scale applications of adsorption are the regeneration and reusability. To evaluate the durability of MnO_2_ NPs, regeneration and reuse studies were conducted at the optimized conditions of MG adsorption over five consecutive cycles. After each run, and prior to reuse, MnO_2_ NPs were collected by centrifugation, washed three times with bidistilled water and ethanol, dried at 80 °C for 2 h, and introduced in the next run.

## Results and discussions

### Characterization of MnO_2_

#### UV–vis characterization

**Fig. 1:**
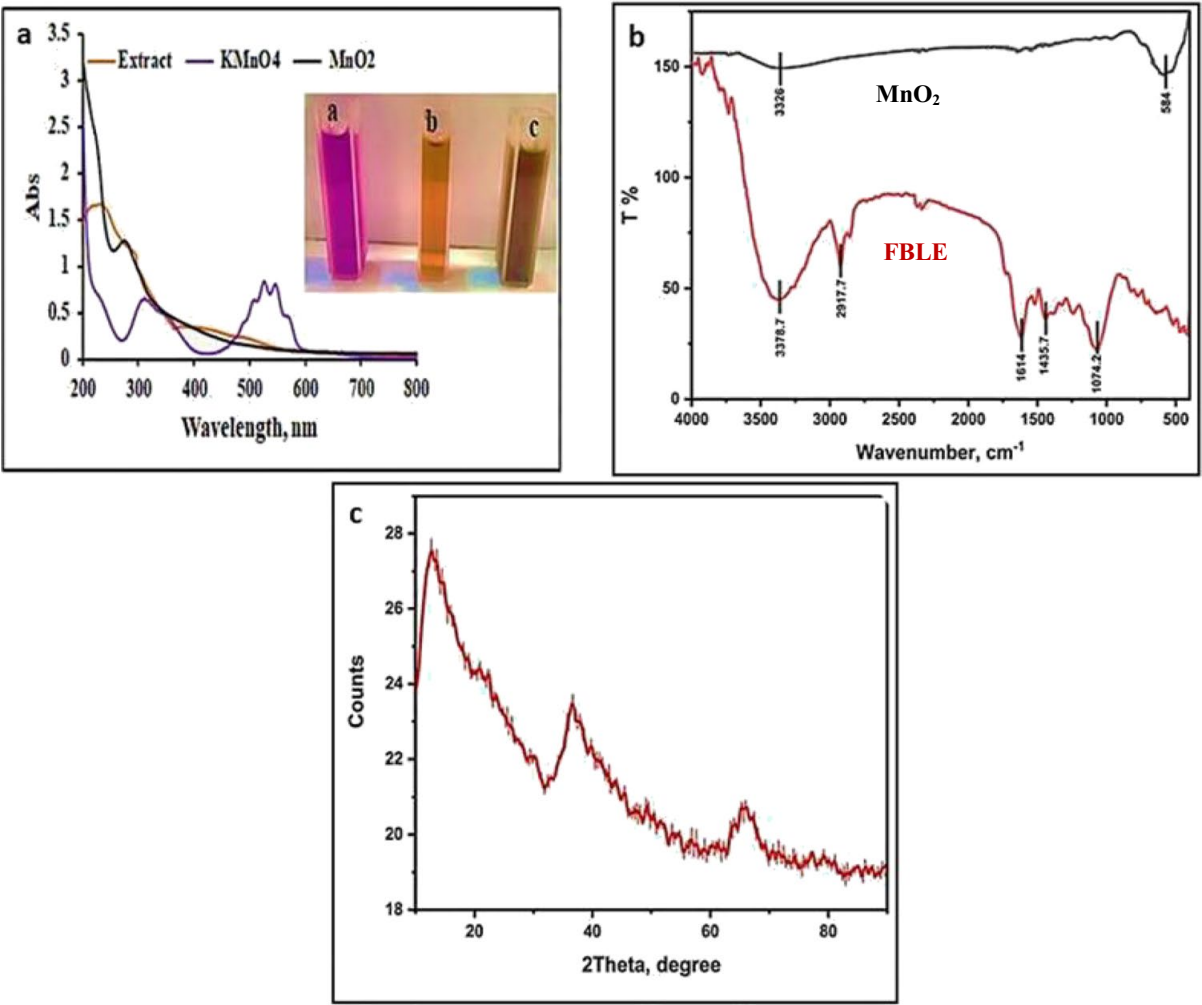
**a** UV–vis spectra of FBLE, potassium permanganate solution, and produced MnO_2_ NPs [inset: color change due to the formation of MnO_2_ NPs], **b** FTIR spectra of FBLE and the biosynthesized MnO_2_ NPs, and **c** XRD pattern of the biosynthesized MnO_2_ NPs.

Bioreduction of manganese ions by phytochemicals present in the FBLE was demonstrated by observing the color shift from pale brown to dark brown (Fig. 1a insert). As a result, the production and stability of NPs in an aqueous solution are validated by the UV–vis spectrum from 200 to 800 nm. Figure 1a shows the UV–vis spectra of FBLE, potassium permanganate solution, and the MnO_2_ NPs that were generated. Aqueous potassium permanganate solution shows two distinct absorption peaks at 310 nm and 530 nm. The polyphenolic content of the FBLE is linked to a characteristic peak at 231 nm. Due to surface plasmon resonance, the aqueous mixture of MnO_2_ NPs appears dark brown and has a pronounced absorption peak at 285 nm (Dessie et al. 2020).

#### FTIR

Phytochemicals in FBLE reduce Mn salt and stabilize the formed MnO_2_ NPs. Thus, useful insights into the chemical composition of the extract and the surface functional groups of MnO_2_ NPs were obtained with FTIR spectroscopy in the wavenumber of 400–4000 cm^−1^. FTIR spectra of FB and the obtained MnO_2_ NPs are shown in (Fig. 1b). The FB exhibits several FTIR absorption bands at 3378.7, 2917.7, 1614, 1435.6, and 1074.2 cm^−1^; these are assigned to OH bond stretching vibration, CH vibration, aromatic C = C bond stretching, bending frequency of methylene group, and C–O stretching, respectively (Abdel-Aziz et al. 2020). Nonetheless, due to OH bond stretching of adsorbed H_2_O molecules, MnO_2_ NPs show only a very broad and less intense absorption band at 3326 cm^−1^ and a more intense band at 584 cm^−1^, which is characteristic of the O–Mn–O bond (Dessie et al. 2020). The absence of any other bands indicates the purity of the sample.

#### XRD

The XRD analysis was utilized to figure out the phase structure and the crystallite size of the biosynthesized MnO_2_ sample as shown in (Fig. 1c). Three large peaks can be seen in the XRD pattern at 2*θ* of 12.745°, 37.627°, and 65.5° which can be indexed as (110), (211), and (002) planes, respectively. All of the diffraction peaks were easily attributed to MnO_2_ in its pure tetragonal phase (COD Card no. 90–16,667) with lattice parameters (*a* = *b* = 9.815, *c* = 2.847) and space group I 4/m (87). The high purity of monophasic MnO_2_ was indicated through the absence of any additional peaks related to impurities. The mean crystallite size of the produced MnO_2_ NPs was calculated as 5.2 nm using Debye–Scherrer’s formula (Khataee et al. 2015).

#### FESEM and EDAX

**Fig. 2:**
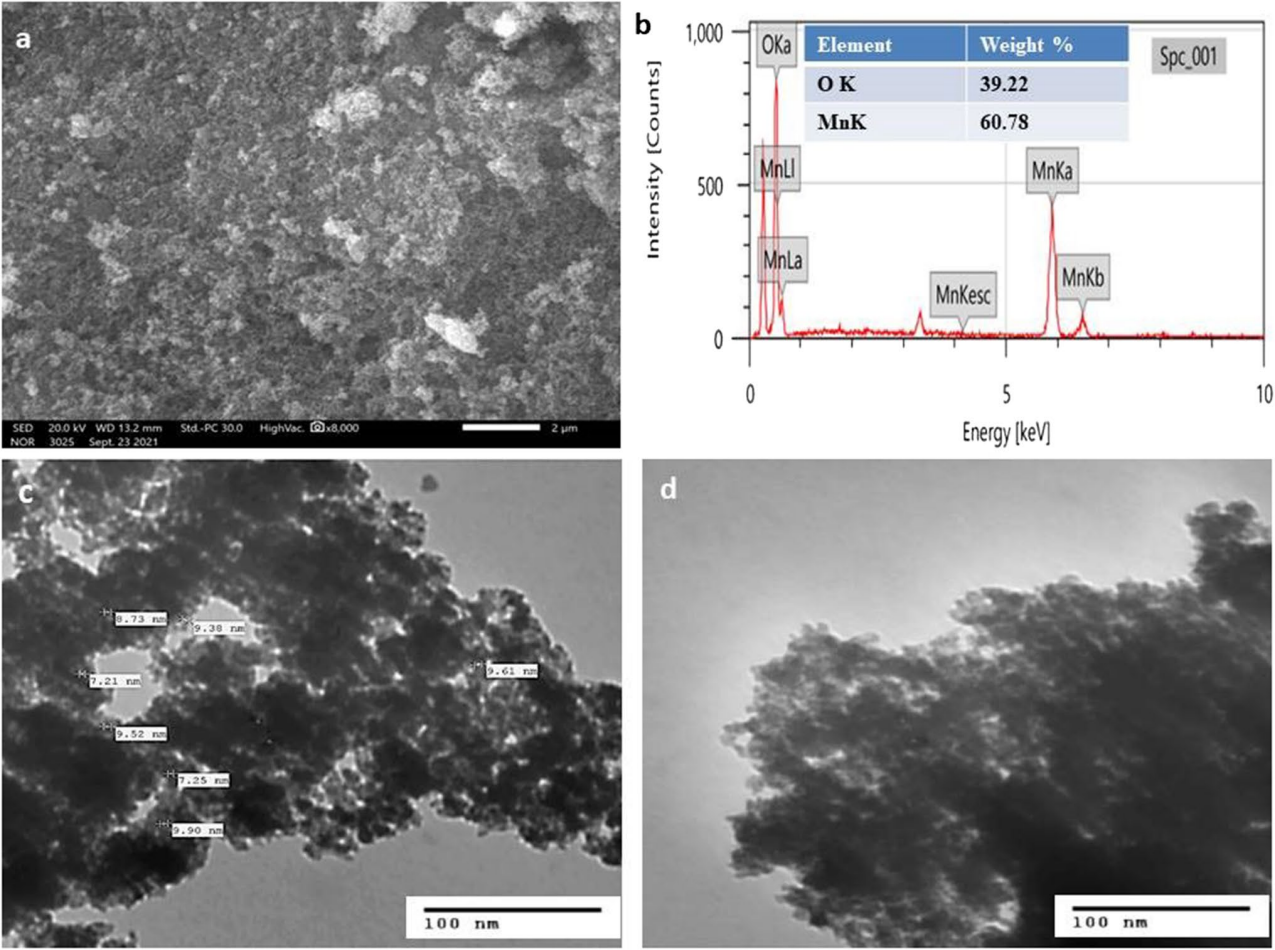
FESEM image (**a**), EDAX spectrum (**b**), and TEM images (**c, d**) of the biosynthesized MnO_2_ NPs

FESEM image was used to examine the morphological features of the biosynthesized MnO_2_ sample (Fig. 2a)**.** This image of MnO_2_ shows aggregations with rough spherical and randomly oriented particles developed with FB aqueous extract. Aggregation of nanoparticles occurred probably due to polarity, electrostatic attraction, high surface energy and usually during the synthesis of nanoparticles in water (Madhumitha et al. 2019). A good adsorption performance for dye removal was expected because of their smaller size and increased surface area. The chemical composition of MnO_2_ NPs was identified by the EDAX technique as shown in Fig. 2b. The EDAX profile shows two strong signals for Mn and O with a molar ratio of 60.78% Mn and 39.22% oxygen. There are no additional peaks in the spectrum, indicating that the sample has been treated to be pure.

#### TEM

The morphology and particle size of MnO_2_ NPs were tested by TEM. The images (Fig. 2c and 2d) showed the agglomerated, extremely small spherical-shaped particles with particle sizes ranging from 7 to 9 nm. This matches well with the XRD results confirming the creation of MnO_2_ NPs through the bioreduction of metal ions by phytochemicals in FB. Moreover, the size reduction can be attributed to the capping action of the active organic compounds in the extract that limited the particle growth (Dessie et al. 2020).

#### BET analysis

**Fig. 3:**
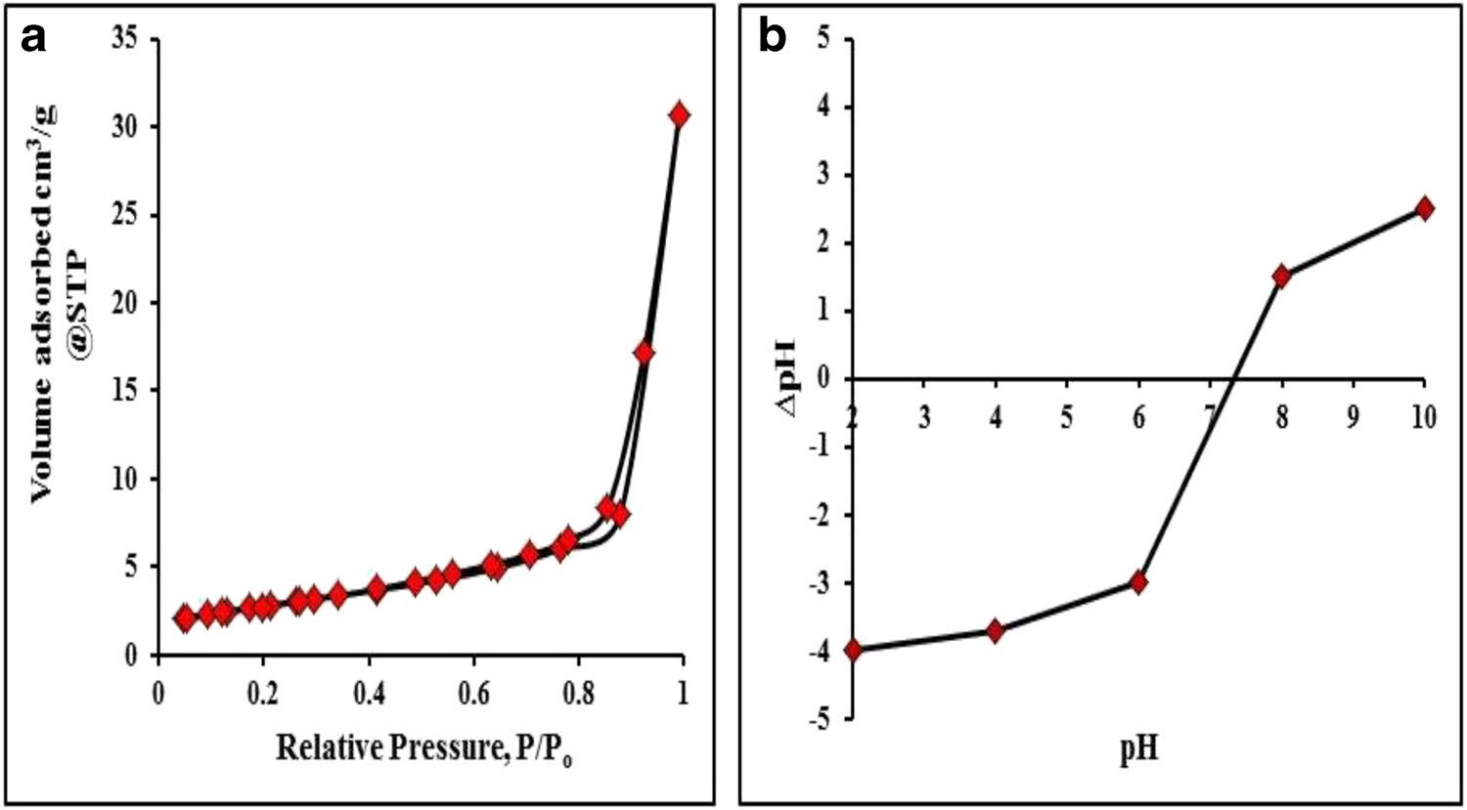
**a** The N_2_ adsorption/desorption isotherm and **b** PZC of MnO_2_ NPs.

The measurement of sorption at the gas/solid interface is required in many fundamental and applied studies of the nature and behavior of solid surfaces. The Barrett, Joyner, and Halenda (BET) method, which utilizes gas molecule sorption to calculate solid surface areas, is frequently used in surface research. The N_2_ adsorption/desorption isotherms of MnO_2_ NPs are shown in Fig. 3a. Type IV isotherms that are frequently associated with mesoporous surfaces were confirmed in the MnO_2_ sample. In addition, the hysteresis loop that arises in the multilayer range of adsorption is typically linked to capillary condensation in mesopores. The test sample’s hysteresis loops are most commonly type H_2_ loops. The sample has a substantial surface area (*S*_BET_ = 149.676 m^2^/g) and a mean pore diameter of 18.959 nm, which is compatible. The BJH theory also yielded a cumulative surface area of 83.8698 m^2^/g, a cumulative pore volume of 0.6685 cm^3^/g, and an average pore width of 3.8396 nm. Pores with a radius of less than 102.11 nm had a total pore volume of 0.7094 cm^3^/g when measured at 0.9905 relative pressure. These results are relatively high for MnO_2_ NPs and confirm the mesoporous structure of the tested sample. Similar results were obtained by Abuzeid et al. (2019). Upon these BET results, the green synthesized MnO_2_ NPs is expected to be a good adsorbent for organic and inorganic contaminants and may be applied in environmental remediation.

#### PZC

Results are shown in Fig. 3b which indicate that the PZC of MnO_2_ NPs is 7.3. At pH lower than the PZC (pH < 7.3), the adsorption of excess H^+^ causes the net surface charge of MnO_2_ NPs to be positive. MnO_2_ NPs have a great ability to adsorb anionic species in this condition. However, at pH > 7.3, the net surface charge of MnO_2_ NPs is negative due to the desorption of H^+^. In this situation, MnO_2_ NPs become suitable for the adsorption of cations like MG dye.

### Optimization of MG adsorption conditions by MnO_2_ NPs

#### Solution pH

**Fig. 4:**
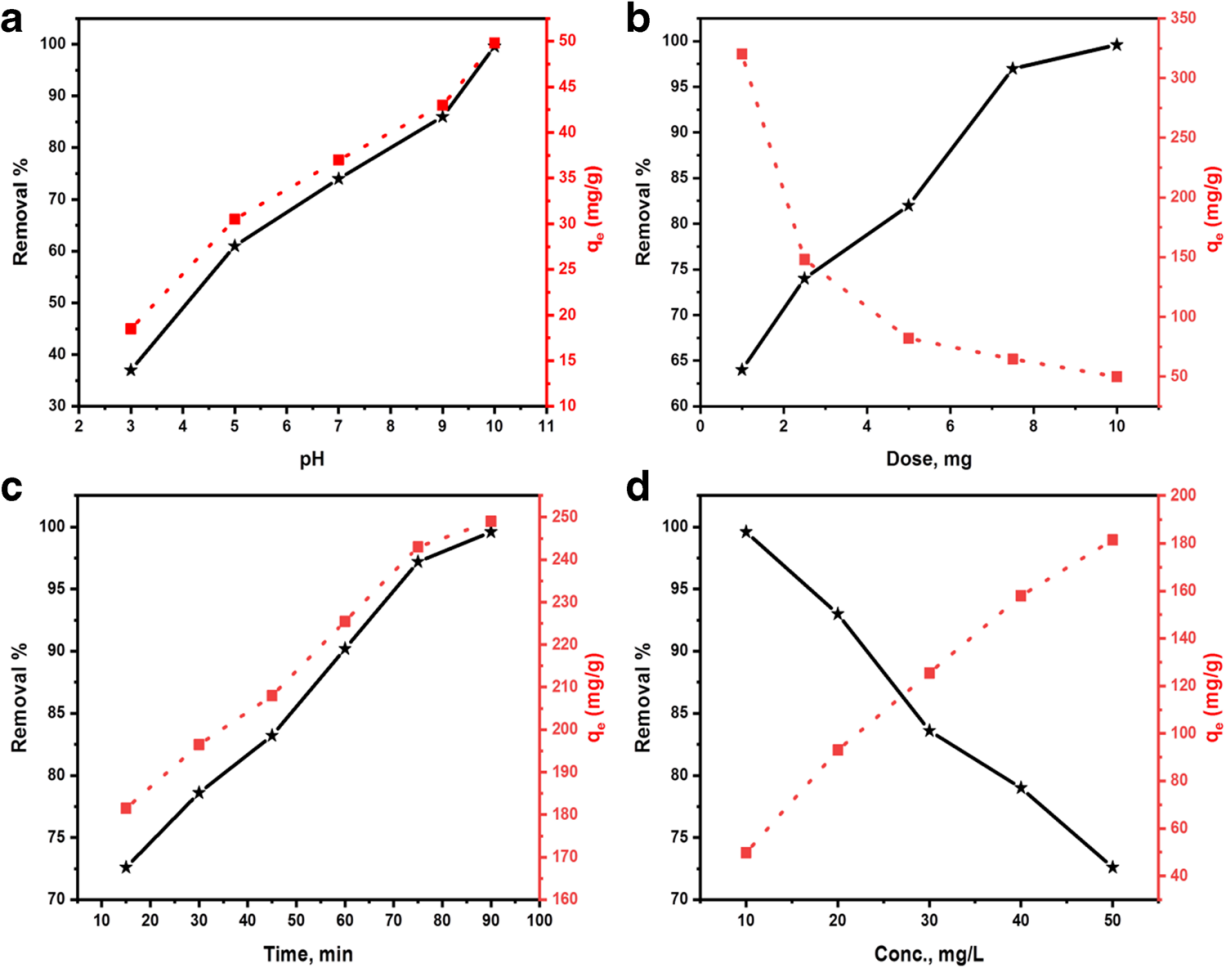
Effects of **a** pH, **b** MnO_2_ NPs dosage, **c** contact time, and **d** initial MG concentration on the MG adsorption process

The pH of the adsorbent and the adsorbing species is critical to the adsorption process because it affects the number of electrostatic charges on both (Eltaweil et al. 2020). It was necessary to test MG adsorption throughout a pH range of 3–10 to find the ideal pH value. According to Fig. 4a, MnO_2_ NPs with different pH levels were able to remove MG from the system. The rate of MG removal rose in direct proportion to pH, reaching a maximum of more than 99.6% at pH 10. The MG *R%* increased from 37% to 99.6% and the MnO_2_ capacity for MG adsorption increased from 18.5 to 49.8 mg/g when pH was raised from 3 to 10. By controlling the pH and PZC of the solution, metal oxides can be used to remove MG from the solution (Guo et al. 2020). The enhanced adsorption of the cationic MG dye at pH values higher than PZC of MnO_2_ (pH > 7.3) is most likely due to the negative surface charges of MnO_2_ NPs induced by deprotonation processes. A pH of 7.3 or lower reduces the adsorption process because positive charges on the MnO_2_ surface electrostatically oppose MG molecules. Other studies have found that high pH has a positive effect on the sorption of MG (Ahmad et al. 2021; Al-Aidy and Amdeha 2021; Gao et al. 2019; Guo et al., 2020). The dye solution’s color and absorption spectra remain stable over a pH range of 3–12 (Fan et al. 2021). Therefore, the following experiments were carried out at pH 10.

#### MnO_2_ NPs dose

For cost-effective application, a batch adsorption system necessitates applying an optimum dosage of suitable adsorbent for maximum adsorbate removal (Al-Aidy and Amdeha 2021). MnO_2_ NPs dosage was studied from 0.001 g to 0.01 g at a fixed concentration of 10 mg/L of MG solution. Figure 4b presented the results of the experiment. Increasing MnO_2_ NPs mass from 0.001 g to 0.01 g resulted in a 64% to 99.6% increase in MG removal efficiency, suggesting that MnO_2_ NPs loading boosted the effectiveness of MG removal (Dehghani et al. 2015; Gao et al. 2019; Nazmara et al. 2020). On the other hand, equilibrium adsorption capacity decreased from 320 to 49.8 mg/g due to the unsaturation of adsorption sites at a greater adsorbent dosage for a given MG concentration (Gao et al. 2019; Nazmara et al. 2020). Moreover, a further increase in dosage beyond 0.01 g did not affect the adsorption capacity of the adsorbent for MG. Thus, an adsorbent dosage of 0.01 g was selected for further experiments.

#### Adsorption time

Practically and economically, the adsorption time plays a significant role in water treatment. The examination of this parameter can set aside money and energy accounts in the case of industrial-scale adsorption processes (Al-Aidy and Amdeha 2021). Figure 4c shows the results of tests on the adsorption of MG by MnO_2_ across periods ranging from 15 to 90 min. By increasing the contact time, MG removal efficiency increased from 72.6% to 99.6%. Also, the adsorption capacity of MnO_2_ NPs for MG increased from 181.5 at 15 min to 249 at 90 min. Several previous studies have shown that the MG adsorption rate increases as the contact time increases (Ahmad et al. 2021; Al-Aidy and Amdeha, 2021; Fan et al. 2021; Gao et al. 2019; Guo et al. 2020). The collected data show that MG adsorption onto MnO_2_ NPs is fairly rapid at first, with around 72.6% of MG molecules adsorbed after 15 min, and then gradually slows down until reaching equilibrium at ~ 90 min. This shows that MnO_2_ NPs have excellent adsorption performance with high adsorption energy. The quick MG adsorption at the start of the process is explained by the rising number of active sites on the MnO_2_ NPs surface as well as the increased gradient of MG concentration in solution and the adsorbent. Laterally, the slowdown in the adsorption of MG was linked to a decrease in free adsorption sites and a strong attraction between MG molecules on the solid and the fluid (Guo et al. 2020). Similar trends were observed by other researchers (Abuzeid et al. 2019; Eltaweil et al. 2020; Guo et al. 2020; Madhumitha et al. 2019). There is no change in the removal efficiency after 90 min. Thus, it was chosen as the equilibration time for further experiments.

#### Initial MG concentration

The initial MG concentration has an effect on the adsorption at interfaces between the aqueous adsorbate and solid adsorbent (Gao et al. 2019). A constant 10 mg dosage of MnO_2_ NPs was used to test the effect of starting MG concentrations, which ranged from 10 to 50 mg/L. The results in Fig. 4d clearly show that the elimination of MG is highly dependent on the concentration at which it is introduced into the system. This decreases from 99.6% to 72.66% when the starting concentration of MG is increased from 10 to 50 mg/L. When adsorbent performance decreased as a result of saturation, it was most likely owing to the presence of dye monolayers on its surface (Belhajjia et al. 2021). Due to an increase in the interaction between MG molecules in the aqueous phase and the surface of MnO_2_ NPs when the initial MG concentration increased from 10 to 50 mg/L, adsorption capacity (*q*_e_, mg/g) increased from 49.8 to 181.5 mg/g.

### Adsorption isotherms

Adsorption isotherms are functional correlations between the concentration of MG in the MG/MnO_2_ NPs interface and the concentration of MnO_2_ NPs in the equilibrium solution at a certain temperature. These isotherms can be used to optimize an adsorption system and provide insight into the affinity of the adsorbent (Gao et al. 2019). Langmuir, Freundlich, Temkin, and Dubinin-Radushkevich (D-R) isotherm models were applied to the experimental data to reveal the mutual association between equilibrium concentration and adsorption capacity. The models’ linear equations and their parameters are shown in Table S1 in the supplementary material.

**Fig. 5:**
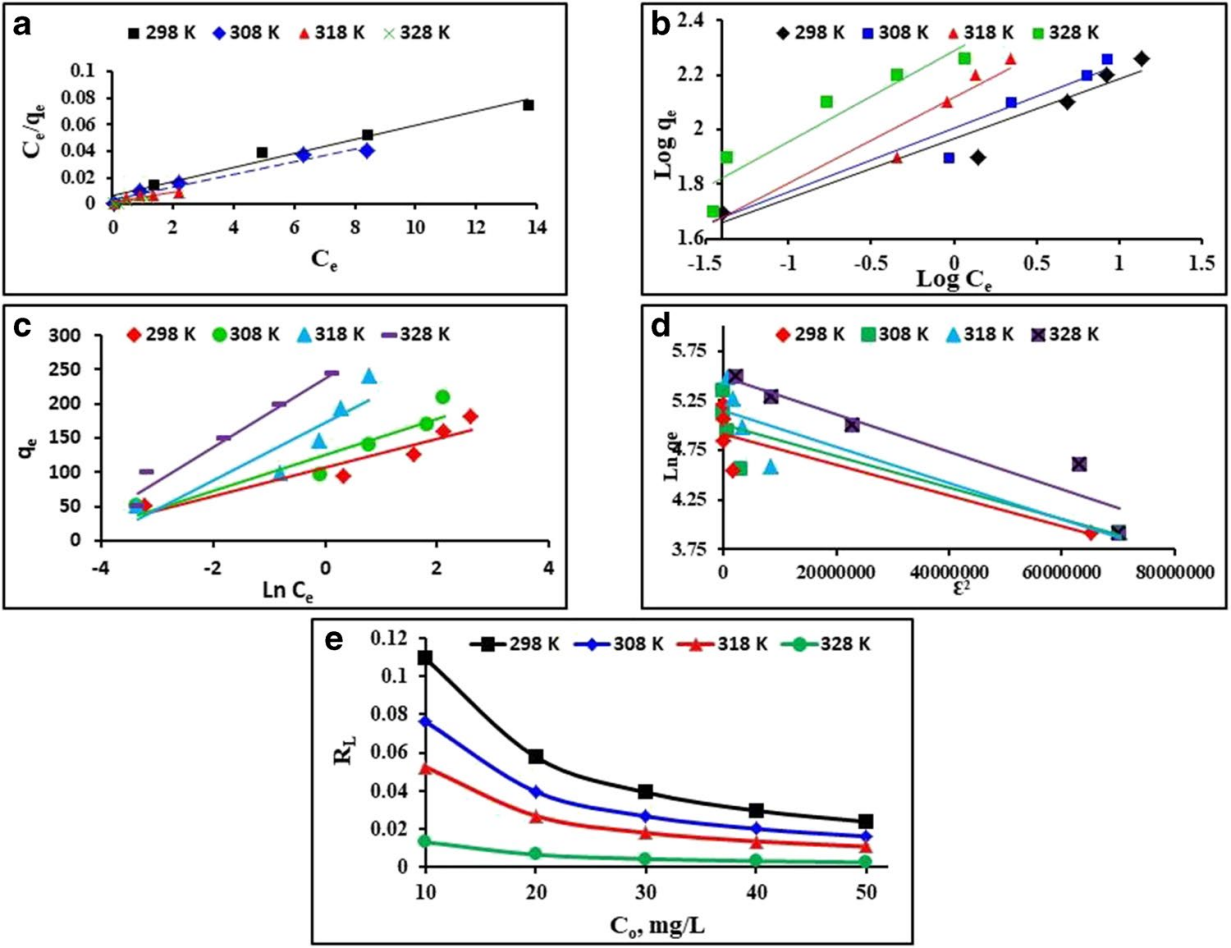
Linear plots of **a** Langmuir, **b** Freundlich, **c** Temkin, and **d** D-R for MG adsorption onto MnO_2_ NPs and **e** variation of adsorption intensity (*R*_L_) with MG initial concentrations at the temperatures 298, 308, 318, and 328 K

For MG adsorption at 298, 308, 318, and 328 K at a variety of MG concentrations ranging from 10 to 50 mg/L linearized Langmuir, Freundlich, Temkin, and D-R linear plots are shown in Fig. 5a, 5b, 5c, and 5d, respectively. The constants for each adsorption isotherm model were used to determine the surface properties and affinity of MnO_2_ NPs for MG (Table 1).

**Table 1:** Parameters of the adsorption isotherm models of MG onto MnO_2_ NPs

T, K	Langmuir			Freundlich			Temkin		D-R		
	*R*^2^	*q*_m_	*K*_L_	*R*^2^	*n*	*K*_F_	*R*^2^	*B*_T_	*R*^2^	*K*_ad_	*q*_s_
298	0.973	188.68	0.815	0.933	4.55	92.385	0.890	21.01	0.776	2 × 10^−8^	136.71
308	0.967	212.77	1.21	0.943	4.26	101.042	0.887	26.41	0.774	1 × 10^−8^	150.16
318	0.89	277.78	1.8	0.925	3.18	131.160	0.834	42.02	0.789	2 × 10^−8^	173.16
328	0.994	270.27	7.4	0.888	3.00	195.119	0.965	50.22	0.895	2 × 10^−8^	243.23

Langmuir model assumes a homogeneous monolayer of MG molecules on the surface of MnO_2_ NPs. As shown in Fig. 5a and Table 1, the high correlation coefficient *R*^2^ values (0.973, 0.967, 0.89, and 0.994 at 298, 308, 318, and 325 K, respectively) indicate that the model simulates the adsorption process well. The nature of adsorption can be evaluated by calculating Langmuir equilibrium parameter *R*_L_ using Eq. (3) (Fan et al. 2021), where *R*_L_ indicates that the adsorption process is irreversible (*R*_L_ = 0), linear (*R*_L_ = 1), favorable (0 < *R*_*L*_ < 1), or unfavorable (*R*_L_ > 1):

3 $${R}_{L}=1/\left(1+{K}_{L}{C}_{o}\right)$$

Figure 5e depicts the connection between the *R*_L_ and the initial MG solution concentration. All *R*_L_ values varied from 0.0023 to 0.1093, indicating that the adsorption process is favorable and efficient at all tested temperatures. Further, the computed Langmuir maximum adsorption capacities ranged from 188.68 to 277.78 mg/g that are greater than those of certain previously published studies (Giri et al. 2022; Mahadevan et al. 2022; Mansa et al. 2016; Pandian et al. 2017).

Freundlich model applies for heterogeneous surfaces and assumes the formation of a multilayer of MG on the MnO_2_ NPs surface. The lower *R*^2^ values (0.888–0.943), as shown in Table 1 and Fig. 5b, indicate that Langmuir model is better to describe the adsorption process over the investigated temperature range. The sorption process is favorable and more heterogeneous as implied by the Freundlich values of 1 < *n* < 10 and 1/*n* < 1, respectively. It is noteworthy that the Freundlich adsorptive capability, as it indicated by *K*_F_ values, is generally lower than that of Langmuir (*q*_m_).

The Temkin equation was also used to fit the data as shown in Fig. 5c and Table 1. The Temkin constants *K*_T_ and *B*_T_ were calculated using the intercepts and slopes of linear connections between *q*_e_ and ln *C*_e_. This model does not fit well the experimental results since *R*^2^ values are even lower than Langmuir and Freundlich models. However, it is possible that MG adsorption onto MnO_2_ NPs was beneficial because of the high Temkin constants B_T_ (21.01–50.22). Table 1 shows that *B*_T_ increased as the temperature rise, indicating endothermic adsorption (Melhi et al. 2022).

D-R modeling was used to verify the experimental results. Unlike in the Langmuir model, here, the uniformity of the surface and the consistency of the sorption potential are absent. The plots of ln *q*_e_ vs. Ɛ^2^ created straight lines at all temperatures, and the resulting *q*_s_ and *K*_ad_ constant values are shown in Table 1. The *R*.^2^ values of D-R linear fit are much lower than all above mentioned models meaning that it cannot fit the results. The mean sorption energy, E, which can be calculated from Eq. (4), is the free energy required to transport one mole of solute from infinity to the adsorbent surface (Wang 2020)

4 $$E=\left(1/\surd 2{\mathrm{K}}_{\mathrm{ad}}\right)$$

When 8 < *E* < 16 kJ/mol, the sorption is considered chemical, and it is physical when *E* ˂ 8 kJ/mol (Wang 2020). The predicted *E* values ranged from 5 to 7.071 kJ/mol at all investigated temperatures meaning that the adsorption process is probably physisorption. Conclusively, Langmuir model is the best to fit the data among all four applied models. For MG adsorption from aqueous solutions, Langmuir equation is used by various researchers (Abuzeid et al. 2019; Ahmad et al. 2021; Eltaweil et al. 2020; Guo et al. 2020; Madhumitha et al. 2019; Yousefi et al. 2021)**.**

### Kinetic study

**Fig. 6:**
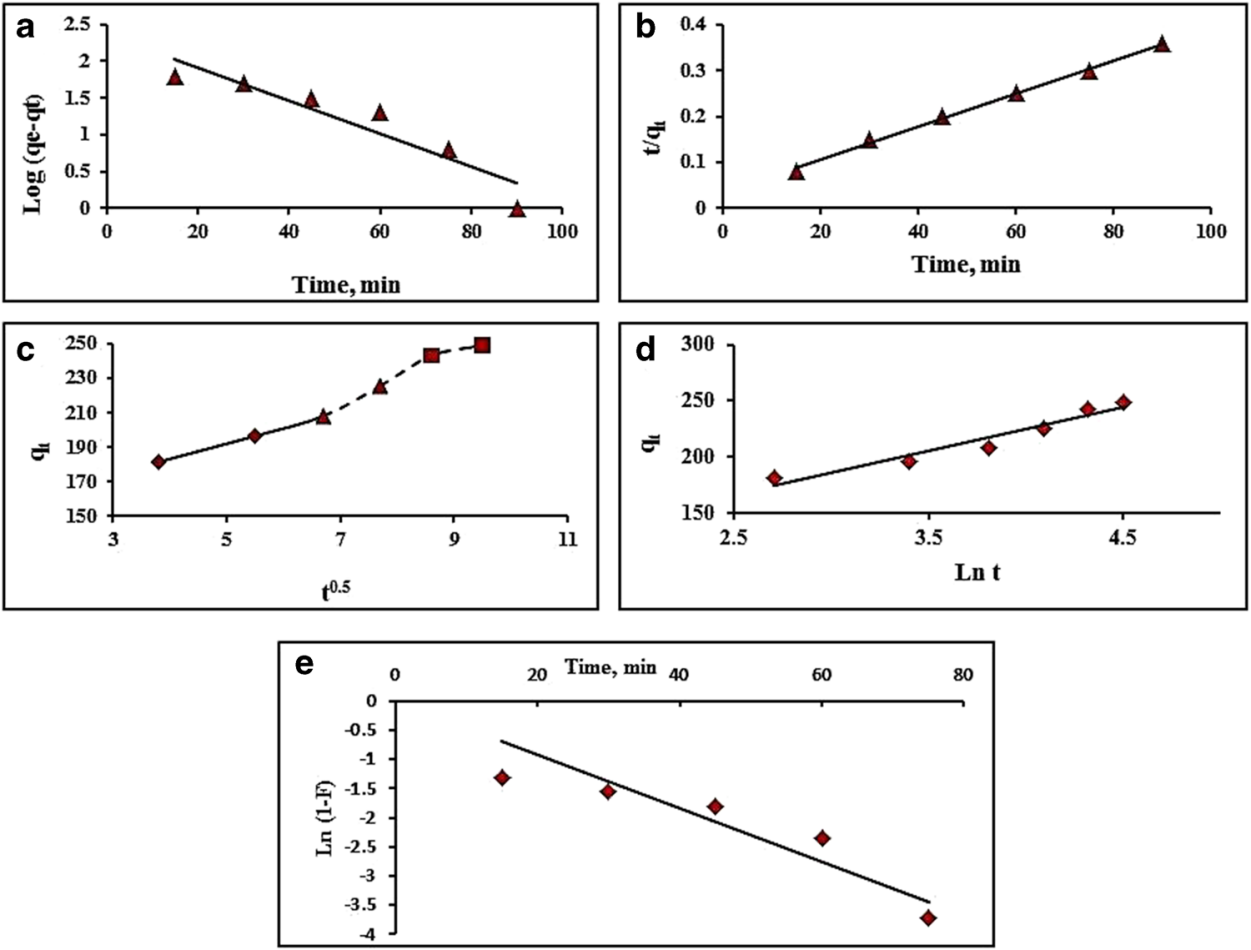
Linear curves of **a** pseudo-first order, **b** pseudo-second order, **c** intraparticle diffusion, **d** Elovich, and **e** Liquid film diffusion kinetic models of MG adsorption process at 298 K

Adsorption kinetics can provide data on the adsorption process's capacities and rates (Eltaweil et al. 2020). The order of interactions between adsorbate and adsorbent has been studied using a variety of kinetic models. The experimental results at optimum conditions (volume, 50 mL; MG initial conc, 50 mg/L; MnO_2_ NPs mass, 0.01 g; and temperature, 25 °C) were tested using pseudo-first order, pseudo-second order, intraparticle diffusion, Elovich, and liquid film diffusion models (Table S2 in supplementary material). Figure 6a–e depict the kinetic results, and the *R*^2^ and model constant values were collected in Table 2. The pseudo-second order model (*R*^2^ = 0.9971) is the best fit for the data compared to other models. Similar findings have been made by other researchers (Eltaweil et al. 2020; Choudhary et al. 2020; Fan et al. 2021; Guo et al. 2020; Mahadevan et al. 2022; Yousefi et al. 2021).

**Table 2:** Calculated parameters for MG adsorption kinetics

Model	Parameters	
Pseudo-first order	*R*^2^	0.8764
	*K*_1_	0.052 min^−1^
	*q*_e_	236.21 mg/g
Pseudo-second order	*R*^2^	0.9971
	*K*_2_	0.0004 g/(mg.min)
	*q*_e_	277.7 mg/g
Intraparticle diffusion	*R*^2^	0.976
	*K*_i_	12.556 mg/(g.min^1/2^)
Elovich	*R*^2^	0.9363
	*α*	232.68 mg/(g.min)
	*β*	0.026 g/mg
Film diffusion	*R*^2^	0.8071
	*K*_fd_	0.0459 min^−1^

The intraparticle diffusion model was studied by graphing the *q*_t_ vs. t^0.5^ relationship in Fig. 6c, which shows the effect of intraparticle diffusion on adsorption. Fluid flow, film diffusion, and plateau region were all represented by the three portions of this curve (Madhumitha et al. 2019). Figure 6c shows that the line does not cross through the origin under the conditions tested. This suggests some boundary layer control and that intraparticle diffusion is not the determining factor in sorption rates.

The experimental results were also represented using the Elovich equation (Table 2). Surface active sites on a solid are thought to vary in character and so have different chemisorption activation energies, according to Elovich’s relation (Mahadevan et al. 2022). As shown in Fig. 6d, the *q*_t_ vs. ln t plot was used to derive the *α* and *β* Elovich coefficients, which are shown in Table 2. The *R*^2^ value of 0.9363 shows that the adsorption is probably chemisorption. The high value of *α* indicates a fast starting chemisorption rate (initial adsorption rate), but the low value of *β* indicates a decreased adsorbent surface for MG adsorption (Mahadevan et al. 2022).

An adsorbate’s diffusion from the bulk of the solution to the adsorbent's surface may also influence the adsorption process’ rate (Zhou et al. 2017). This can be tested by applying the liquid film diffusion model to the results (Table 2 and Fig. 6e). As can be seen from the *R*^2^ value (0.807) of the linear plot of ln (1-F) vs. t, this model’s applicability is limited (Zhou et al. 2017), and the adsorption process is not controlled by the diffusion of MG molecules via the liquid film surrounding MnO_2_ NPs adsorbent.

### Thermodynamic studies

#### Influence of temperature

**Fig. 7:**
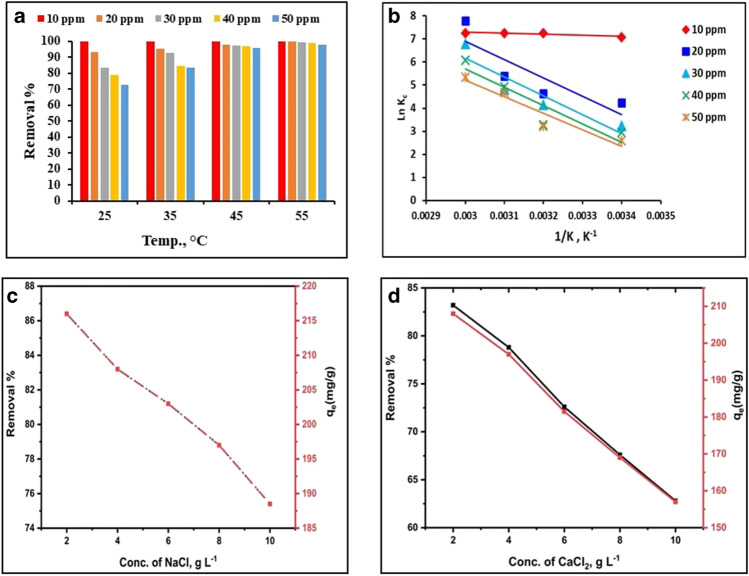
**a** Influence of temperature, **b** Van’t Hoff curve, and **c**, **d** the respective effect of NaCl and CaCl_2_ on the performance of MnO_2_ NPs.

Thermal conditions were thought to play an important role in adsorption capacity and reaction speed. The effect of temperature on MG adsorption by MnO_2_ NP is shown in Fig. 7a**.** The capacity and effectiveness of adsorption were both enhanced by the rise in temperature. The mobility of MG and its interaction with the active sites on MnO_2_ NPs surface may be responsible for this. Furthermore, the higher the initial MG concentration, the more MG molecules are available for adsorption at higher temperatures. The adsorption process is endothermic, according to previous studies (Abou-Gamra and Ahmed 2015; Ahmad et al. 2021; Al-Aidy and Amdeha 2021; Altintig et al. 2021).

#### Thermodynamic parameters

 Thermodynamic parameters such as enthalpy change (ΔH°), entropy change (ΔS°), and Gibbs free energy change (ΔG°) can be calculated using equilibrium data from isotherm investigations. These parameters support the adsorption nature. The following Eqs. (5–8) are used to calculate these thermodynamic parameters (Mahadevan et al. 2022):

5 $${K}_{c}={C}_{s}/{C}_{e}$$

6 $$\Delta G^{\circ}=-RT\mathrm{ln}{K}_{c}$$

7 $$\Delta G^{\circ}=\Delta \mathrm{H}^{\circ}-\mathrm{T}\Delta \mathrm{S}^{\circ}$$

8 $$\mathrm{ln}{K}_{c}=\left(\Delta \mathrm{S}^{\circ}/\mathrm{R}\right)-\left(\Delta \mathrm{H}^{\circ}/\mathrm{RT}\right)$$

where *C*_e _(mg/L) and *C*_s_ (mg/g) are the respective equilibrium MG concentrations in solution and adsorbed onto MnO_2_, *K*_c_ (L/g) is the thermodynamic equilibrium constant, *R* (8.314 J/mol/K) is the universal constant, and *T* (K) is the absolute temperature. Results are given in Table 3.

**Table 3:** Results of thermodynamic analysis for MG adsorption

Conc, mg/L	Temp, K	ΔG°, kJ/mol	ΔH°, kJ/mol	ΔS°, kJ/(mol.K)
10	298	− 17.543	3.913	0.072
	308	− 18.263		
	318	− 18.983		
	328	− 19.703		
20	298	− 9.96	66.03	0.255
	308	− 12.51		
	318	− 15.06		
	328	− 17.61		
30	298	− 8.181	67.809	0.255
	308	− 10.731		
	318	− 13.281		
	328	− 15.831		
40	298	− 7.054	65.755	0.244
	308	− 9.494		
	318	− 11.934		
	328	− 14.374		
50	298	− 3.381	59.497	0.211
	308	− 5.491		
	318	− 7.601		
	328	− 9.711		

Slope and intercept were used to derive ΔH° and ΔS° values from Van’t Hoff's linear plot (ln *K*_c_ versus 1/T) (Fig. 7b). Endothermicity and the physical nature of MG adsorption onto MnO_2_ NPs are indicated by the positive ΔH° values of 3.91 to 67.81 kJ/mol that is less than 80 kJ/mol. A positive ΔS° (0.211 kJ/mol) indicates that the entropy is increased because of the redistribution of energy between MG and MnO_2_ NPs as a result of adsorption. These physically adsorbed species are thermodynamically stable because of the negative values of ΔG° (− 3.38 to − 19.70) across the whole temperature range. Low temperature changes have a significant impact on the adsorbent as a whole. Similar findings have been reported by other research group (Mohanta et al. 2020a).

### Influence of interfering ions

Dye manufacturing effluent contains large amounts of inorganic cations. An increase in the concentration of these cations induces colloidal instability, which inhibits interactions between dye molecules and the adsorbent (Dong et al. 2021). With the same experimental conditions of 50 mg/L MG concentration, 10 mg MnO_2_ NPs, and 60 min of contact time at 25 °C, the effect of different Na^+^ and Ca^2+^ cation concentrations (2–10 g/L) on MG adsorption over MnO_2 _NPs has been examined. Figures 7c and 7d show the findings. MG removal percentage dropped from 86.4% to 75.4% as well as the adsorption capacity from 216 to 188.5 mg/g when the Na^+^ concentration was raised (Fig. 7c). The MG removal efficiencies decreased from 83.2% to 62.8%, and the MnO_2_ adsorption capacities dropped from 208 to 157 mg/g in the presence of Ca^2+^ (Fig. 7d). This decrease in MG uptake can be explained by the competition between positively charged MG molecules and metal cations for the negatively charged sites on MnO_2_ NPs surface. As a result, adsorption was reduced when Ca^2+^ was present because the divalent Ca^2+^ ions blocked more binding sites than the monovalent Na^+^ ions (Mittal et al. 2020).

### Effect of competing cationic dyes on adsorption

**Fig. 8:**
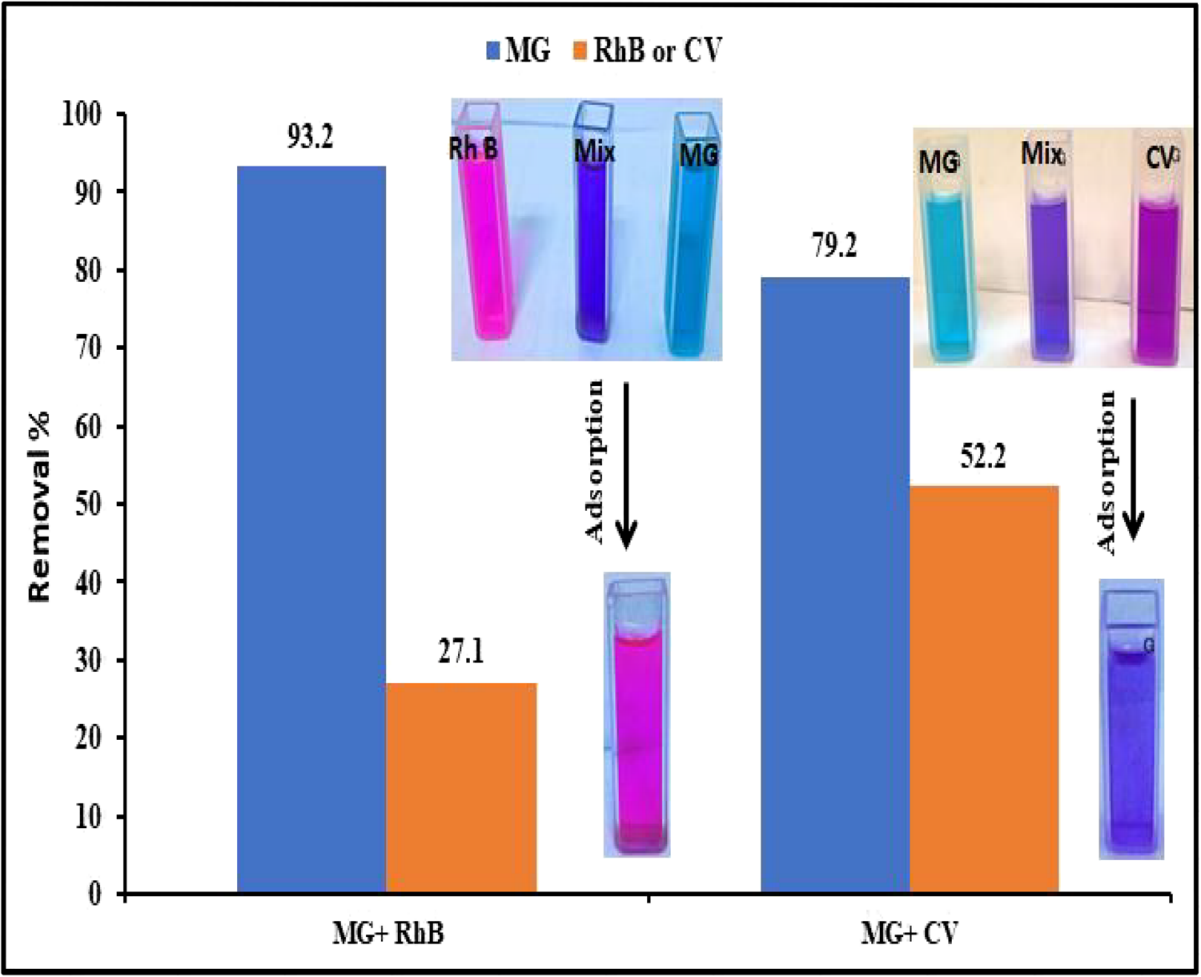
Adsorption efficiency of MG by MnO_2_ NPs in presence of RhB or CV at equal concentrations

To examine the selectivity of MnO_2_ NPs towards MG, its adsorption was conducted in the presence of coexisting cationic dyes such as crystal violet (CV) and rhodamine B (RhB) with the same concentration at optimum conditions. The obtained results were exposed in Fig. 8. The adsorption efficiency of MG by MnO_2_ NPs was found to be substantially higher compared to other dyes, since it was 79.2% in the presence of CV and 93.2% in the presence of RhB. CV and RhB, on the other hand, had adsorption efficiencies of 52.2% and 27.1%, respectively. It was found that RhB’s adsorption steric barrier was too high for the transfer of dye molecules to the adsorbent pores (Mittal et al. 2016). Calculating the separation factor (α) from Eq. (9) was used to assess the selectivity of MG adsorption in the presence of competing CV and RhB dyes:

9 $$\mathrm{\alpha }={\mathrm{K}}_{\mathrm{d}}\left(\mathrm{MG}\right)/{\mathrm{K}}_{\mathrm{d}}\left(\mathrm{dye}\right)$$

where *K*_d_ refers to the affinity of MG towards adsorption and is called the coefficient of distribution which can be calculated from Eq. (10):

10 $${\mathrm{K}}_{\mathrm{d}}=\left[\left({\mathrm{C}}_{0}-{\mathrm{C}}_{\mathrm{e}}\right)/{\mathrm{C}}_{\mathrm{e}}\right]\left(\mathrm{V}/\mathrm{W}\right)$$

The calculated separation factors of MG in the presence of CV and RhB are 3.5 and 36.6, respectively (Table S3 in supplementary material). The high *α* values suggest higher selectivity of MG adsorption by MnO_2_ NPs.

### Reusability and stability of MnO_2_ adsorbent

**Fig. 9:**
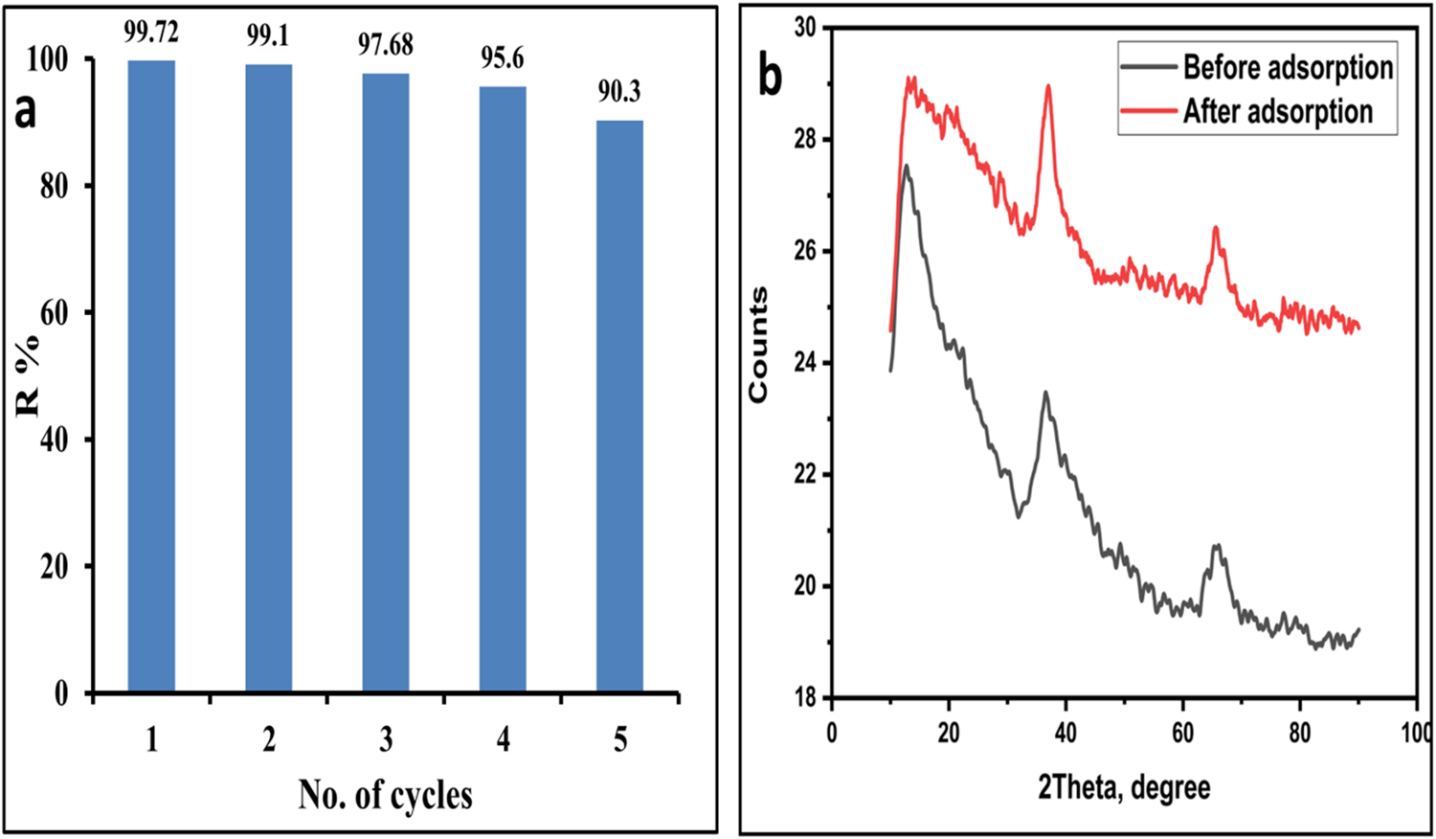
**a** Removal efficiency of MG by MnO_2_ NPs after five cycles and **b** MnO_2_ XRD spectra before and after MG adsorption.

Partially large-scale applications require adsorbent regeneration and stability. Figure 9a reflects the study of these parameters at the best conditions for MG adsorption by MnO_2_ NPs. A centrifuge was used to separate MnO_2_ NPs from the reaction mixture, which was then rinsed with bidistilled H_2_O and ethanol and dried at 80 °C before being used in the next set. After the fifth run, the percentage of MG elimination reduced marginally from 99.72% to 90.33%, according to the data. XRD was used to analyze the structural stability of MnO_2_ NPs after the fifth adsorption experiment (Fig. 9b). Stability of MnO_2_ NPs has been confirmed by its crystallographic structure, as demonstrated by its consistent XRD pattern. As a result, MnO_2_ is an adsorbent that is stable, reusable, resilient, and separable and may be used to effectively remove dyes.

### Mechanism of MG adsorption onto MnO_2_ NPs

**Fig. 10:**
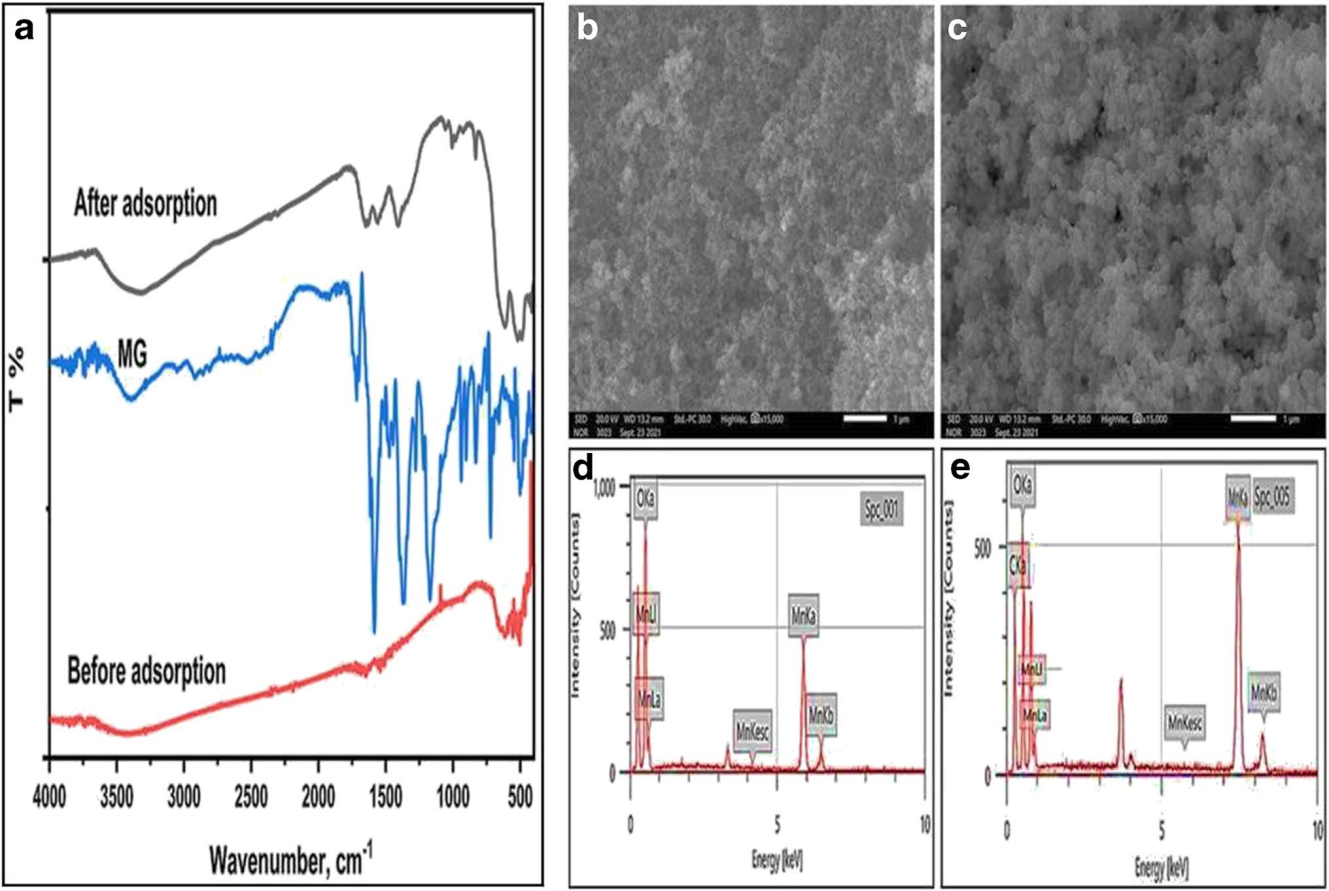
**a** FTIR spectra of MG, MnO_2_ NPs, and MG loaded MnO_2_ NPs, **b, c** SEM images of MnO_2_ NPs before and after MG adsorption, and **d, e** EDAX profiles of MnO_2_ NPs before and after MG adsorption.

The mechanism of MG elimination was studied using FTIR, SEM, and EDAX techniques. Figure 10a shows the FTIR spectra of MnO_2_ NPs before and after MG adsorption. The NH stretching and bending vibrations were assigned from the two distinct bands at 3321 and 1651 cm^−1^ in the MnO_2_ NPs spectrum, which clearly illustrates the interaction of MnO_2_ NPs with cationic MG groups (Guo et al. 2020). Multiple weak peaks appeared between 1006 and 832 cm^−1^ in MnO_2_ NPs spectrum after MG adsorption. These peaks are attributed to bending vibrations of the C-H bonds of MG methyl groups indicating its adsorption on MnO_2_ NPs surface. Stretching vibrations of the C-N group at 1405 cm^−1^ were used to further examine the strong binding between MG and MnO_2_ NPs. Further confirmation of MG adsorption is the appearance of the characteristic benzene ring vibration peak at 1600 cm^−1^ after adsorption. The lower intensity of this peak compared with pure MG spectrum may indicates the π-π interaction between benzene rings after adsorption. In addition, a lower wavenumber at 520 cm^−1^ replaces the typical Mn–O stretching peak at 584 cm^−1^. Electrostatic contact between positively charged MG molecules with the negative OH groups on the surface of MnO_2_ NPs above PZC is indicated by these characteristics (Kaur et al. 2021).

**Fig. 11:**
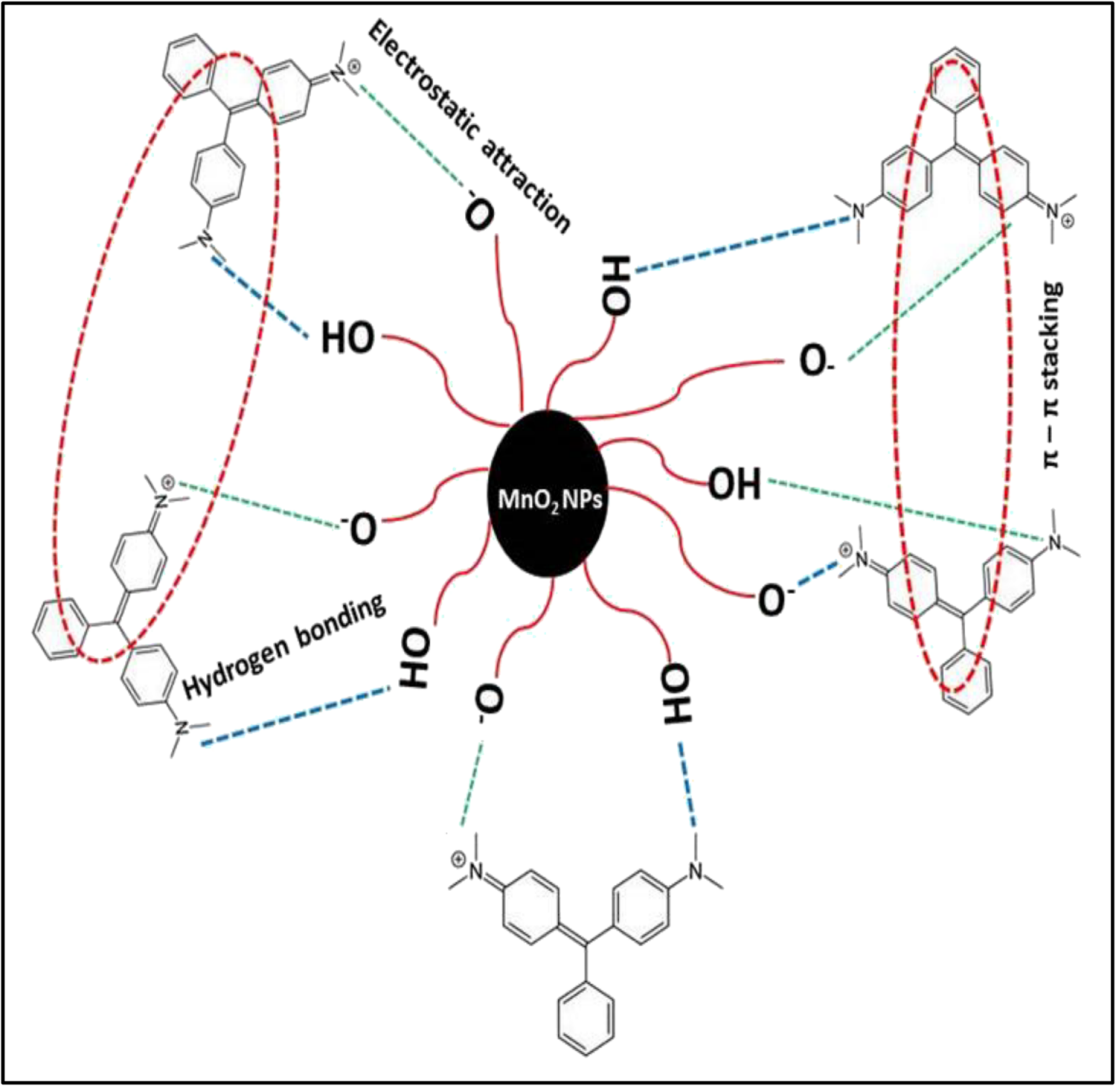
Schematic illustrations of the electrostatic attraction and hydrogen bonding between MnO_2_ NPs and MG dye

In addition, SEM images of MnO_2_ NPs before and after the MG adsorption are recorded at room temperature with the same magnifications (Fig. 10b and c). It can be seen that the adsorbent particles were roughly spherical and randomly oriented before MG adsorption (Fig. 10b). SEM image after adsorption in Fig. 10c clearly shows MG loading on MnO_2_ NPs since particles became denser and more compact with no change in shape. The EDAX spectra of MnO_2_ NPs before and after adsorption were compared to study the MG adsorption mechanism (Fig. 10d and e). MnO_2_ NPs show a carbon signal after adsorption of MG, which confirms the successful loading of MG. Adsorption by MnO_2_ NPs was most likely due to the H-bonding between MnOH and N atoms in alkaline medium, the π-π stacking of aromatic rings, and the electrostatic attraction between the positive MG nitrogen atoms and the negative OH surface groups of MnO_2_ NPs as shown in Fig. 11.

#### Comparison with other adsorbents

The optimum conditions and the adsorption capacity of MG by some previously reported adsorbents are summarized in Table 4. The as-synthesized MnO_2_ showed a higher capacity for MG adsorption than most of the reported adsorbents.

**Table 4:** Comparison of MG adsorption conditions and capacities onto different adsorbents

Adsorbent	pH	Mass (g/L)	*q*_max_ (mg/g)	Time (min)	Temp. (°C)	Ref
CoO NPs	7.0	0.12	238.10	120	-	Mohanta et al. (2020a)
ZnO-AC^a^	7.0	0.1	303.03	60	45	Altintig et al. (2021)
CuO NPs	8.0	0.5	178.89	120	-	Kumar et al. (2017)
NiO NPs	8.0	0.5	189.0	120	-	Kumar et al. (2017)
NiO NPs	7.0	0.25	87.72	80	-	Mohanta et al. (2020b)
Fe_2_O_3_ NPs	9.0	1	82.64	120	25	Kaur et al. (2021)
Ag NPs	7.8	0.1	64.52	-	30	Pandian et al. (2017)
Silica NPs	10	0.1	24.81	200	-	Mansa et al. (2016)
MnO_2_ NPs	9.0	0.45	-	30	-	Kumar et al. (2014)
TiO_2_ NPs	-	0.1	63.08	30	30	Abou-Gamra and Ahmed (2015)
Ag NPs	9.0	2.0	268.82	40	50	Dawodu et al. (2019)
Chinese fan palm seed biochar	7.0	1.0	21.41	1440	35	Giri et al. (2022)
PisAC^b^	7.0	4.0	76.92	30	-	Mahadevan et al (2022)
CMC/GG/GO^c^	6.5	0.1	17.6	30	-	Naeini et al. (2022)
Chitosan/Fe_3_O_4_	7	2	55.86	210	-	Mashkoor et al. (2022)
Fe_3_O_4_/MgO/AC^a^	-	-	2474	-	45	Guo et al. (2020(
MnO_2_ NPs	10.0	0.01	188.68	90	25	**Present study**
			212.77		35	
			277.78		45	

^a^AC, activated carbon; ^b^*Pistacia vera* L. shell-based active carbon, ^c^cellulose/guar gum/graphene oxide.

Particularly, our adsorbent was synthesized by a low-cost and eco-friendly method utilizing FBLE. Also, it has superior proprieties such as small particle size, high surface area, richness in surface acidic oxygen groups, and mesoporosity with large pore volume which provide a greater number of available active sites and improve interaction probabilities for adsorption. Moreover, mesoporous adsorbents are favored due to the ability of dye molecules to penetrate their channel textures. Additionally, the synthesized MnO_2_ nano adsorbent has selectively adsorbed MG dye. Thus, it is promising for selective adsorption of MG from a variety of aqueous environments.

## Conclusion

Here, ficus leaves extract was used as reducing and capping agent to green synthesize mesoporous MnO_2_ NPs from the permanganate salt without adding extra harmful chemicals. The resulting MnO_2_ NPs were then applied to remove MG from aqueous solutions by adsorption. MG at a concentration of 10 mg/L was almost completely removed (99.72%) within a short time (90 min) using a tiny mass of MnO_2_ NPs (0.01 g) at room temperature (25 °C) in alkaline medium (pH 10). Pseudo-second-order (*R*^2^ = 98%) and Langmuir (*R*^2^ = 99.7%) models suit the experimental results well. Experiments at different temperatures under optimal conditions resulted in Langmuir maximum adsorption capacities of 188.68, 212.77, 277.78, and 270.27 mg/g at 25, 35, 45, and 55 °C, respectively. Further, the MG adsorption process was both endothermic (positive ΔH°) and thermodynamically spontaneous (negative ΔG°). The removal efficiency results in the presence of coexisting cationic dyes (CV and RhB); monovalent (Na^+^) and divalent (Ca^2+^) ions confirmed the preferential adsorption of MG onto MnO_2_ adsorbent. MnO_2_ stability was proofed by the slight decrease in the removal efficiency (9%) after five consecutive sorption cycles. Moreover, results indicated that the adsorption was likely physical (adsorption energy < 8 kJ/mol, ΔG° < 0, and ΔH° < 80 kJ/mol) and probably occurred through electrostatic interaction, π-π interaction, and hydrogen bonding. Finally, the cost-effective and eco-friendly biosynthesized MnO2 with high adsorption capacity and stability can be scaled up to an industrial scale and efficiently applied for the removal of MG from water and wastewater.

## Supplementary Information

Below is the link to the electronic supplementary material.

Supplementary file1 (DOCX 48 kb)

## Data Availability

The datasets generated during and/or analyzed during the current study are available from the corresponding author on reasonable request (I.M.A.H).
