# Supplementary material for: Ficus-mediated green synthesis of manganese oxide nanoparticles for adsorptive removal of malachite green from surface water
Source: Environ Sci Pollut Res Int. 2022 Nov 17;30(10):28144–61. doi: 10.1007/s11356-022-24199-8 (PMC9995432; doi:10.1007/s11356-022-24199-8)
Supplement: Supplementary file 1 — Supplementary file1 (DOCX 48 kb) [file 11356_2022_24199_MOESM1_ESM.docx]

**Ficus-mediated green synthesis of manganese oxide nanoparticles for adsorptive removal of malachite green from aqueous solutions**

Ibrahem Mohamed Abouzeid Hasan*, Hassan M. A. Salman, Olfat M. Hafez

Chemistry Department, Faculty of Science, South Valley University, Qena 83523, Egypt

*Corresponding author: Tel +201006753052, E-mail: [hasan111167@gmail.com](mailto:hasan111167@gmail.com) (I.M.A .Hasan), ORCID: 0000-0001-9329-2804.

**S2. Materials and** **procedures**

**S2.1. Instrumentation for the characterization of the biosynthesized MnO_2_ NPs**

The biosynthesized MnO_2_ NPs were preliminary characterized using UV-vis spectrophotometer (PG Instruments, model T80, UK) using quartz cells of 1 cm pathlength, and in the 200 to 800 nm wavelength range. To evaluate the possible role of phytochemicals in the synthesis of NPs, FTIR spectra were recorded in the range of 400-4000 cm^-1^ using KBr pellet method (Shimadzu FTIR, Kyoto, Japan). To investigate the phase structure and crystallite size of the biosynthesized sample, X-ray diffraction spectra were recorded at room temperature using a powder diffractometer (Brucker D8 Advance, Germany with Cu Kα radiation source, λ = 1.5406Å and 2θ in the range (10-90°). The mean size of the prepared samples crystals was calculated from Scherrer equation (D = 0.9λ / (β cos θ)), where D is the average crystallite size (nm), λ is the X-ray wavelength used (λ = 1.54056 Å), θ is the diffraction angle and β is the full width at half the maximum of the diffraction peak in radians. The morphology, size, and chemical structure of the synthesized NPs was examined using a field-emission scanning electron microscope (Jeol, JSM-IT200, Japan) attached with energy dispersive X-ray from the same company which was operated at 20 kV accelerating voltage, 13.2 mm working distance and probe current of 0.1 nA. TEM analysis was carried out to show the crystallography, morphology, and composition of the synthesized samples, using Jeol Jem-1230 operating at accelerating voltage of 200 KV. BEL SORP-MAX analyzer (MicrotracBEL, Japan) was used to measure the specific surface areas from N_2_ adsorption/desorption data by application of the multipoint BET (Brunauer-Emmet-Teller). BJH theory was adopted for measuring the mean pore diameter.

**S2.2. Study of point of zero charge (PZC)**

The pH drift method (Worch 2021) with some modifications was employed to determine the point of zero charge (PZC) of MnO_2_ NPs. Briefly, each 50 mL of NaCl aqueous solution (0.01 M) was mixed with fixed MnO_2_ NPs loading 2.0 g/L in five 250 mL reagent bottles. Then, the initial pH of the solution (pH_i_) in each bottle was adjusted by using NaOH (0.1 M) or HCl (0.1 M) solutions at pH range (2 - 10) followed by agitation with the sorbent for 48 h to attain the equilibrium. Finally, the final pH of the solutions (pH_f_) was measured and ΔpH was calculated (ΔpH = pH_i_ – pH_f_) and plotted versus pH_i_. The point of null value variation (ΔpH = 0) is the PZC. Calibration of the pH meter at pH 4, 7, and 10 was done before each run.

**S3. Results and discussions**

**S3.3. Adsorption isotherms**

**Table S1** Isotherm models and their parameters used in this study.

| **Model** | **Equation** | **Parameters** | **Ref.** |
| --- | --- | --- | --- |
| **Langmuir** | C_e_/q_e_ = (1/K_L_.q_m_) + (C_e_/q_m_) | q_e_ (mg/g): equilibrium adsorption capacity  q_m_ (mg/g): maximum adsorption capacity  K_L_ (L/mg): Langmuir constant; adsorption affinity  C_e_ (mg/L) : equilibrium adsorbate concentration in solution | (Tang et al. 2022) |
| **Freundlich** | log q_e_ = (𝟏/𝒏) log C_e_ + log K_F_ | K_F_ (mg/g) /(mg/ L)^1/n^ represents adsorption capacity  1/n (dimensionless): Freundlich constant provides adsorption intensity | (Tang et al. 2022) |
| **Temkin** | q_e_=B_T_ ln K_T_ + B_T_ ln C_e_ | K_T_ (L/g): Temkin adsorption potential  B_T_ (J/mol): Temkin constant | (Melhi et al. 2022) |
| **(D–R)** | ln q_e_ = ln q_s_ − K_ad_ε^2^  ∈ = RT ln (1 + 1/c_e_) | q_s_ (mg/g): theoretical isotherm saturation capacity  K_ad_ (mol^2^/J^2^): Dubinin–Radushkevich isotherm constant  ∈ (J/mol): Polanyi potential which is related to the equilibrium concentration  R (8.314 J/mol/ K): universal gas constant  T (K): temperature | (Melhi et al. 2022) |

**S3.4. Kinetic study**

**Table S2** Kinetic models used in this study.

| **Model** | **Equation** | **Parameters** | **Ref.** |
| --- | --- | --- | --- |
| **Pseudo-first-order** | Log (q_e_−q_t_) = log q_e_ – (k_1_/2.303) t | q_t_ ( mg/g): amount of adsorbate adsorbed at time t  k_1_ (min^-1^): pseudo-first-order rate constant | (Lagergren 1898) |
| **Pseudo-second-order** | q_t_ = (1/k_2_q_e_^2^) + (1/q_e_) t | k_2_ [g/(mg.min)]: pseudo-second-order rate constant | (Ho and McKay 1999) |
| **Intraparticle-diffusion** | q_t_ = k_i_ t^0.5^ + C | k_i_ [mg/(g.min)^0.5^)]: intraparticle diffusion rate constant | (Weber et al. 1963) |
| **Elovich** | q_t_ = (1/β) ln αβ + (1/β) lnt | *α* [mg/(g.min)]: the initial adsorption rate  *β* (g/mg): parameter related to the extent of surface coverage and activation energy of Elovich equation. | (Roginsky et al. 1934) |
| **Liquid film diffusion** | ln (1−F) = −k_fd_ t | F = q_t_/q_e_: fractional attainment of equilibrium | (Boyd et al. 1947) |

**Table S3** Parameters of adsorption selectivity in binary mixtures.

| **Binary mixture** | **Dye** | **K_d_** | **α** |
| --- | --- | --- | --- |
| MG/CV | MG | 19.1 | 3.5 |
|  | CV | 5.46 |  |
| MG/RhB | MG | 68.14 | 36.6 |
|  | RhB | 1.86 |  |

**References**

Boyd GE, AW Adamson and LS Myers Jr. J Am Chem Soc )1947( The Exchange Adsorption of Ions from Aqueous Solutions by Organic Zeolites. II. Kinetics 69 :2836-2848 https://doi.org/10.1021/ja01203a066

Ho YS, McKay G (1999) Pseudo-second order model for sorption processes. Process Biochem 34: 451 465. https://doi.org/10.1016/S0032-9592(98)00112-5

Lagergren S (1898) Zur theorie der sogenannten adsorption geloster stoffe. Kungliga svenska vetenskapsakademiens. Handl 24:1-39.

Melhi S, Algamdi M, Alqadami AA, Khan MA, Alosaimi EH (2022) Fabrication of magnetically recyclable nanocomposite as an effective adsorbent for the removal of malachite green from water. Chem Eng Res Des 177:843-854.‏ <https://doi.org/10.1016/j.cherd.2021.11.028>

Roginsky, S, Zeldovich YB (1934). The catalytic oxidation of carbon monoxide on manganese dioxide. Acta Phys Chem USSR 1:2019.

Tang R, Hong W, Srinivasakannan C, Liu X, Wang X, Duan X (2022) A novel mesoporous Fe-silica aerogel composite with phenomenal adsorption capacity for malachite green. Sep Purif Technol 281:119950.‏ <https://doi.org/10.1016/j.seppur.2021.119950>

Weber TW, Chakravorti, RK (1974). Pore and solid diffusion models for fixed‐bed adsorbers. AIChE J 20:228-238. https://doi.org/10.1002/aic.690200204

Worch E (2021) Adsorption Technology in Water Treatment: Fundamentals, Processes, and Modeling. Berlin Boston: De Gruyter. <https://doi.org/10.1515/9783110715507>
